# Prognostic Value of Serum (1→3)-β-D-Glucan Levels in Patients with Candidemia Stratified by Compliance with Candida Bundle: A Multicenter Retrospective Cohort Study (2016–2023)

**DOI:** 10.1007/s11046-025-00999-7

**Published:** 2025-09-22

**Authors:** Hidemasa Akazawa, Shinnosuke Fukushima, Toshie Higuchi, Tomoko Miyoshi, Yasuhiro Nakano, Koji Iio, Yukinobu Akamatsu, Yuto Haruki, Yoshitaka Iwamoto, Shuichi Tanaka, Shun Fujisato, Soichiro Ako, Hideharu Hagiya

**Affiliations:** 1 Department of General Medicine, Okayama University Graduate School of Medicine, Dentistry and Pharmaceutical Sciences, Okayama, Japan; 2 Department of Infectious Diseases, Okayama University Hospital, 2-5-1 Shikata-cho, Kitaku, Okayama, 700-8558 Japan; 3 Department of Bacteriology, Okayama University Graduate School of Medicine, Dentistry and Pharmaceutical Sciences, Okayama, Japan; 4 Department of General Internal Medicine, Okayama Red Cross Hospital, Okayama, Japan; 5 Center for Medical Education and Internationalization, Kyoto University Graduate School of Medicine, Kyoto, Japan; 6 Microbiology Division, Clinical Laboratory, Okayama University Hospital, Okayama, Japan; 7 Department of General Medicine, Tottori Municipal Hospital, Tottori, Japan; 8 Department of Pharmacy, Tsuyama Chuo Hospital, Okayama, Japan; 9 Department of General Medicine, NHO Okayama Medical Center, Okayama, Japan; 10 Department of Pharmacy, Okayama Rousai Hospital, Okayama, Japan

**Keywords:** Candidemia, Prognosis, β-D-glucan, Candida bundle, Endophthalmitis

## Abstract

**Background:**

Candidemia is a severe systemic infection with a high mortality risk. While β-D-glucan (BDG) serves as a diagnostic biomarker, its prognostic value in candidemia, particularly in association with Candida bundle compliance, remains unclear.

**Methods:**

In this retrospective multicenter cohort study, we evaluated 96 patients with candidemia across nine Japanese hospitals between 2016 and 2023. Candida bundle compliance was assessed using five key components: central venous catheter removal within 24 h of diagnosis, appropriate initial antifungal therapy, ophthalmologic examination, follow-up blood cultures until clearance, and antifungal therapy for at least two weeks post-clearance. Analyses stratified patients by serum BDG status (positive/negative) and compliance with the Candida bundle (high: 4–5 points; low: 0–3 points). The primary outcome was 30-day mortality, and the secondary outcome was defined as endophthalmitis incidence.

**Results:**

Of 96 eligible patients with candidemia, 70 (72.9%) were BDG-positive and 26 (27.1%) were BDG-negative. The overall 30-day mortality was 17.7%. Among BDG-positive patients, 15 (21.4%) died, while 2 (7.7%) died in BDG-negative cohorts (*p* = 0.09). Serum BDG positivity demonstrated a statistically significant association with decreased survival rates in the low bundle adherence group (*p* = 0.02), whereas this correlation was not observed among patients in the high-compliance cohort (*p* = 0.66). Endophthalmitis occurred in 25.0% of patients, without significant correlation to serum BDG status. *C. albicans* was associated with a significantly higher incidence of endophthalmitis compared with non-albicans species (45.7% vs. 8.9%).

**Conclusions:**

Serum BDG positivity potentially correlates with worse survival in candidemia, particularly in patients with low bundle compliance. This emphasizes the importance of adherence to standardized Candida management protocols for optimizing patient outcomes.

**Supplementary Information:**

The online version contains supplementary material available at 10.1007/s11046-025-00999-7.

## Introduction

*Candida* species are ubiquitous fungal microorganisms [1], even constituting a substantial proportion of the normal microbiota of human cutaneous surfaces, gastrointestinal system, urogenital tract, and vaginal cavity [2]. They cause opportunistic infections primarily in immunocompromised individuals, including patients with malignant neoplasms, human immunodeficiency virus (HIV)/acquired immunodeficiency syndrome (AIDS), or diabetes mellitus, and those undergoing hemodialysis [3]. Additionally, individuals undergoing immunosuppressive therapy, those who have recently undergone abdominal surgery, as well as older populations are particularly susceptible to these infections.

*Candida* infections primarily manifest as two clinical presentations: superficial candidiasis and invasive candidiasis [4]. The most prevalent form of invasive candidiasis is *Candida* infection of the bloodstream (candidemia), wherein the pathogen can potentially disseminate and infect multiple organ systems, including the hepatic, splenic, renal, cardiovascular, ophthalmologic, skeletal, and central nervous systems [5]. Candidal endophthalmitis represents a significant ophthalmologic complication secondary to candidemia, occurring in approximately 15–25% of patients [6, 7]. The mortality rate of candidemia ranges from 26.5 to 45% [8–10], and its epidemiological incidence has increased during the coronavirus disease-2019 (COVID-19) pandemic [11]. Reflecting this epidemiological trend, the World Health Organization published the Fungal Priority Pathogen List (FPPL) in 2022 [12], wherein *Candida albicans* was classified in the critical priority group, while *Candida tropicalis* and *Candida parapsilosis* were designated to the high-priority category [13]. Additionally, the global emergence of *Candida auris* has been increasingly becoming an emerging concern [14].

The following critical interventions contribute to clinical management and improved outcomes of patients with candidemia: (i) collection of two sets of blood cultures; (ii) early and adequate antifungal therapy; (iii) early source control; (iv) removal of central venous catheters (CVCs) within 24 h of diagnosis; (v) assessment of the clinical efficacy of antifungal therapy between days 3 and 5; (vi) ophthalmologic consultation; (vii) follow-up blood culture testing until candidemia clearance; (viii) optimization of treatment duration; and (ix) step-down oral therapy for patients with favorable clinical course. Candidemia bundles—developed using these evidence-based core management elements [15–18]—have been significantly effective, with high protocol compliance associated with mortality rates reducing from 28 to 18% [16].

Early diagnosis and therapeutic intervention are critical for achieving optimal clinical outcomes in patients with candidemia. Nevertheless, blood culture—the primary diagnostic methodology—exhibits suboptimal sensitivity and requires prolonged detection time; moreover, the results may potentially remain negative until advanced stages of infection [19, 20]. (1→3)-β-D-Glucan (BDG), a conserved fungal cell wall polysaccharide, serves as a complementary diagnostic biomarker for detecting invasive fungal infections [21], which is known to become detectable in serum an average of 2.5 days prior to blood culture positivity [22]. The diagnostic performance of BDG for candidemia demonstrates a sensitivity ranging 55–92%, specificity ranging 60–80%, and a notably high negative predictive value of 97% or higher [4, 23]. Therefore, clinicians typically interpret a negative serum BDG test as a low probability of the disease. However, approximately 20% of clinical patients constitute serum BDG-negative candidemia [24]. While negative BDG results are suggested to correlate with a potentially more favorable prognosis [24], comprehensive investigation of the multifactorial variables in Candida management bundles is lacking. Therefore, in this study, we primarily aimed to elucidate the nuanced interactions among Candida bundle scores, serum BDG results, and outcomes of patients with candidemia. Additionally, we assessed the incidence of endophthalmitis in relation to BDG test results as a secondary objective.

## Methods

### Study Design and Settings

In this retrospective, multi-center observational study, data of participants across nine healthcare facilities located in Okayama, Tottori, and Kagawa prefectures in Japan, encompassing an 8-year surveillance period (from January 2016 to December 2023), were included. Clinical and microbiological data pertaining to patients diagnosed with candidemia were extracted from electronic medical records utilizing standardized methodological protocols.

### Inclusion and Exclusion Criteria

Inclusion criteria encompassed hospitalized patients aged ≥ 18 years with *Candida* species detected in blood cultures. The following patients were excluded: (a) patients with blood culture contamination; (b) patients who died prior to initiating antifungal therapy; (c) those who died within three days of treatment initiation; (d) those who were discharged or transferred from the hospitals without outcome documentation; (e) patients with incomplete data; (f) those without serum BDG measurements; and (g) those without follow-up blood cultures.

### Definitions and Study Protocol

Candidemia was defined as the detection of *Candida* species in at least one blood culture, and nosocomial candidemia was defined as those occurring beyond 48 h after hospital admission [25]. The original candidemia management bundle comprised the following nine components: (1) expeditious removal of CVCs within 24 h of diagnosis; (2) initial selection of appropriate antifungal agent; (3) precise dosing of initial antifungal agent; (4) comprehensive ophthalmological evaluations; (5) continuous blood culture monitoring until candidemia clearance; (6) systematic assessment of clinical efficacy between days 3–5; (7) judicious selection of alternative antifungal therapies; (8) antifungal therapies for a minimum of two weeks following candidemia clearance; and (9) step-down oral therapeutic strategies for patients with favorable clinical progression [17, 18, 26]. To streamline our analysis, we selected five core interventions as critical therapeutic components: (i) expeditious removal of CVCs within 24 h of diagnosis; (ii) initial selection of appropriate antifungal agent; (iii) comprehensive ophthalmological evaluations, (iv) continuous blood culture monitoring until candidemia clearance; and (v) antifungal therapies for a minimum of two weeks following candidemia clearance. The selection of appropriate alternative therapy is inherently patient-specific and was therefore not evaluated in this study. The exclusion of step-down oral therapy from candidemia management protocols potentially enhanced clinical outcomes and reduced mortality [18]. Therefore, oral switch therapy was not included in the study. The initial selection of appropriate antifungal agents was determined in accordance with the clinical practice guidelines established by the Infectious Diseases Society of America [26]. Given the retrospective nature of this study, obtaining comprehensive data on precise antifungal dosages and clinical efficacy assessments proved practically challenging.

We comprehensively evaluated underlying comorbidities, including diabetes mellitus, chronic kidney disease (defined as estimated glomerular filtration rate [eGFR] < 30 mL/min/1.73 m^2^), hemodialysis, malignancy, anticancer pharmacotherapy, and immunosuppressive interventions. Additionally, we assessed candidemia-associated risk factors such as surgical intervention within the preceding month, burn injury, and CVC insertion [17]. The following microbiological data were collected: source of infection, serum BDG values, number of blood culture sets collected, positive blood culture sets, detected *Candida* species, and the physician's determination of potential contamination.

The manufacturers of blood culture devices and culture bottles, in conjunction with the culture duration protocols implemented across participating facilities, are presented in Supplementary Table 1. In accordance with another study, we established the cutoff values for the β-D-glucan assays as follows: 11 pg/mL for the Fujifilm Wako β-Glucan test kit (Fujifilm Wako Pure Chemical, Osaka, Japan) and 20 pg/mL for the Fungitec® G test kit (Nissui Pharmaceutical, Tokyo, Japan) [27]. The Fungitec® G test was performed using either the ES analyzer (Nissui Pharmaceutical, Tokyo, Japan) or the Wellreader SK603 (Seikagaku Corporation, Tokyo, Japan). The Fujifilm Wako assay was performed using LIMUSAVE MT-7500 system (Fujifilm Wako Pure Chemical, Tokyo, Japan) (Supplementary Table 2). Both assays exhibit similar sensitivity (ranging from 0.90 to 0.93) when interpreted using their respective recommended cutoff values; we did not adjust for inter-assay differences. Serum BDG levels were assessed within a window of 3 days from the collection date of the positive blood culture. In patients where serum BDG levels were measured multiple times, the highest value was used for further analysis, as it may represent the peak fungal burden and provide the strongest prognostic signal.

### Outcome Measures and Statistical Analysis

The primary outcome was to assess 30-day overall mortality and its association with serum BDG status, stratified by Candida bundle score (high score: 4–5 points; low score: 0–3 points). The secondary outcome was the evaluation of the incidence of endophthalmitis between these groups. For baseline comparisons, the Mann–Whitney U test was used for continuous variables such as patient age. For categorical variables, the Chi-square test was applied when sample sizes were sufficient (N > 5), while Fisher’s exact test was used when expected frequencies were small. EZR software, a comprehensive graphical user interface for R version 3.5.2, was used to perform log-rank tests. Statistical significance was set as a two-tailed *p* value of < 0.05.

### Ethics Approval

All protocols were conducted in strict adherence to the ethical principles outlined in the Helsinki Declaration of 1975 (as subsequently revised in 2008), and aligned with the established ethical standards of national and institutional human experimentation review committees. Given the retrospective observational design involving patient medical records, informed consent was obtained through a standardized opt-out methodology. Ethical approval for this investigation was obtained from the Okayama University Ethics Institutional Review Board (Approval No. 2404–045).

## Results

**Fig. 1:**
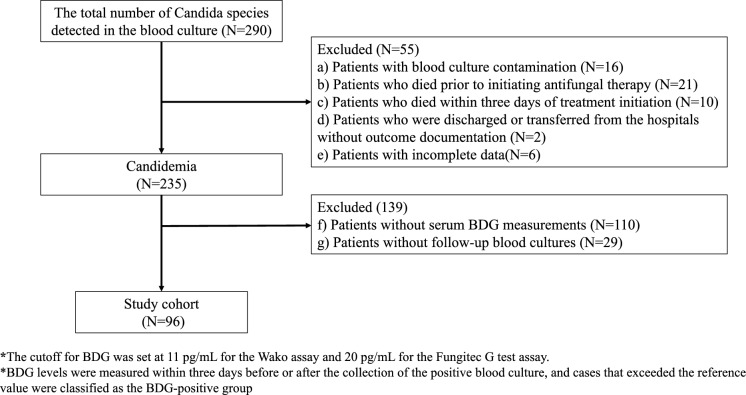
Flowchart depicting the enrolment of participants

During the 8-year study period from 2016 to 2023, a total of 290 patients with *Candida* species isolated from blood cultures were identified. After applying exclusion criteria, 194 patients were excluded, resulting in a final analytical cohort of 96 patients. The patients were excluded as follows: (a) culture contamination (n = 16), (b) death before the initiation of antifungal therapy (n = 21), (c) death within 72 h of treatment initiation (n = 10), (d) discharge before treatment completion (n = 2), (e) incomplete clinical data (n = 6), (f) absence of serum BDG measurements (n = 110), and (g) lack of follow-up blood culture testing (n = 29) (Fig. 1). Of the 96 patients, 26 (27.1%) demonstrated negative BDG assay results.

Table 1 summarizes the baseline demographic and clinical characteristics of the 96 patients. The median age was 72 years (interquartile range [IQR] 57–77), with men comprising 68.8% of the cohort. Nosocomial candidemia cases were predominant, accounting for 93.8% of patients. Serum BDG was measured mostly before/after 0 to 3 days of candidemia onset, with no significant difference in timing distribution between BDG-positive and BDG-negative groups. Predominant underlying comorbidities included malignancies (53.1%), diabetes mellitus (46.9%), and immunosuppressive agents (37.5%). Primary infection sources include CVC-associated candidemia (76.0%), intra-abdominal infections (9.4%), and urinary tract infections (8.3%). Overall compliance with the Candida bundle protocol was substantial, with high adherence scores (4–5 points) observed in 83.3% of patients. The high adherence rates were comparable between serum BDG-positive (84.3%) and serum BDG-negative (80.8%) groups without statistically significant differences (*p* = 0.76).

**Table 1:** Baseline demographics of patients with Candidemia stratified by BDG status

	All patients (N = 96)	BDG Positive (N = 70)	BDG Negative (N = 26)	*p* value
Age, years	72 [57–77]	73 [59–78]	64 [55–75]	0.18
Sex, male, N (%)	66 (68.8)	48 (68.6)	18 (69.2)	1.00
In-hospital onset, N (%)	90 (93.8)	64 (91.4)	26 (100)	0.18
Time from the diagnosis of candidemia to BDG testing, days	1 [0–2]	1 [0–2]	1 [0–2]	1.00
Background condition, N (%)				
Malignancy	51 (53.1)	39 (55.7)	12 (46.2)	–
Diabetes mellitus	45 (46.9)	34 (48.6)	11 (42.3)	–
Immunosuppressive agents	36 (37.5)	22 (31.4)	14 (53.8)	–
Chemotherapy	28 (29.2)	19 (27.1)	9 (34.6)	–
Recent operation	23 (24.0)	16 (22.9)	7 (26.9)	–
Chronic kidney disease	22 (22.9)	14 (20.0)	8 (30.8)	–
Hemodialysis	17 (17.7)	12 (17.1)	5 (19.2)	–
Burn injury	2 (2.1)	2 (2.9)	0 (0)	–
Origin of Candidemia, N (%)				
CV catheter-related	73 (76.0)	52 (74.3)	22 (84.6)	–
Abdominal	9 (9.4)	8 (11.4)	1 (3.8)	–
Urinary tract	8 (8.3)	6 (8.6)	2 (7.7)	–
Primary	5 (5.2)	4 (5.7)	1 (3.8)	–
Other	1 (1.0)	1 (1.4)	0 (0)	–
Candida bundle score, N (%)				
High score (4–5 points)	80 (83.3)	59 (84.3)	21 (80.8)	0.76
5 point	45 (46.9)	38 (54.3)	7 (26.9)	–
4 point	35 (36.4)	21 (30.0)	14 (53.9)	–
Low score (0–3 points)	16 (16.7)	11 (15.7)	5 (19.2)	–
3 point	9 (9.4)	5 (7.1)	4 (15.4)	–
2 point	5 (5.2)	4 (5.7)	1 (3.8)	–
1 point	2 (2.1)	2 (2.9)	0 (0)	–
0 point	0 (0)	0 (0)	0 (0)	–

*BDG, (1, 3)-β-D-glucan; CVCs, central venous catheters

Species distribution analysis revealed *Candida albicans* as the predominant pathogen (44.8%; 43/96), followed by *Candida glabrata* (17.7%; 17/96) and *Candida parapsilosis* (14.6%; 14/96). *Candida guilliermondii* demonstrated significantly higher frequency of negative serum BDG assays compared to other Candida species (*p* = 0.001) (Table 2).

**Table 2:** Distribution of isolated *Candida* species, by BDG status

Candida species, N (%)	All patients (N = 96)	BDG Positive (N = 70)	BDG Negative (N = 26)	*p* value
*C. albicans*	43 (44.8)	33 (47.1)	10 (38.5)	0.50
*C. glabrata*	17 (17.7)	11 (15.7)	6 (23.1)	0.39
*C. parapsilosis*	14 (14.6)	11 (15.7)	3 (11.5)	0.75
*C. tropicalis*	8 (8.3)	8 (11.4)	0 (0)	0.10
*C. guilliermondii*	5 (5.2)	0 (0)	5 (19.2)	0.001
*C. krusei*	5 (5.2)	3 (4.3)	2 (7.7)	0.61
Others*	4 (4.2)	4 (5.7)	0 (0%)	0.57

*BDG, (1, 3)-β-D-glucan. Others include two cases each of *C. famata* and *C. dubliniensis*

**Fig. 2:**
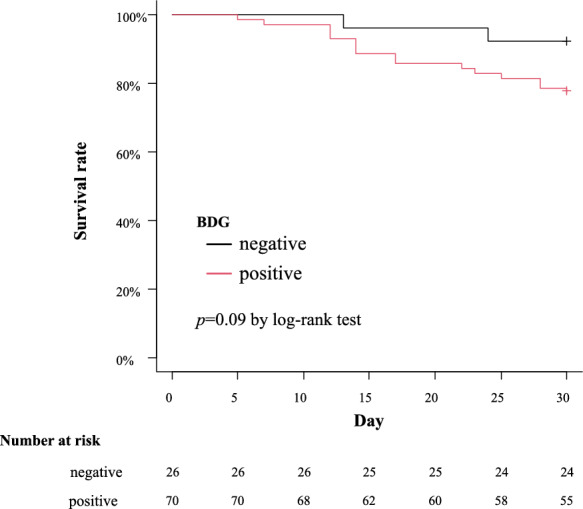
Kaplan–Meier survival curves comparing the prognosis of all patients with candidemia, stratified by β-D-glucan (BDG) status. While not achieving statistical significance by log-rank analysis (*p* = 0.09), the serum BDG-positive group demonstrated a trend toward increased mortality compared to the BDG-negative group (21.4% vs. 7.7%)

**Fig. 3:**
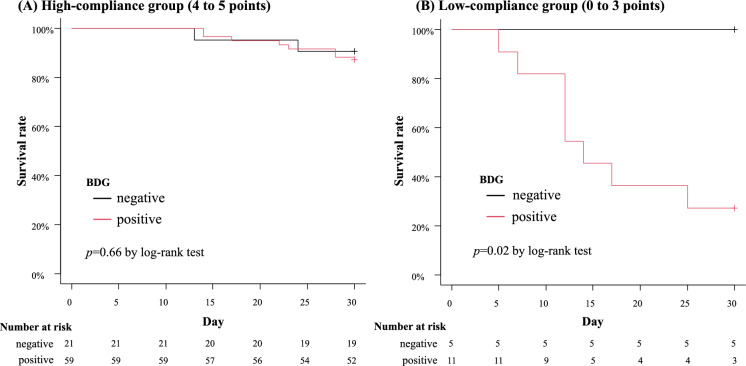
Kaplan–Meier survival curves of patients grouped by Candida bundle score: high-compliance group (**A**) and low-compliance group (**B**). In the high-compliance cohort, serum BDG status showed no significant association with survival outcomes (11.9% vs. 9.5%; *p* = 0.66, log-rank test). However, in the low-compliance cohort, serum BDG positivity correlated significantly with increased mortality (72.7% vs. 0%; *p* = 0.02, log-rank test)

Figure 2 provides the Kaplan–Meier curves comparing 30-day survival between serum BDG-positive and serum BDG-negative groups. The overall mortality rate was 17.7% (17/96). In accordance with serum BDG status, the serum BDG-positive group demonstrated a relatively higher mortality rate than the serum BDG-negative group (21.4% vs. 7.7%; *p* = 0.09, log-rank test). To further explore the impact of serum BDG on survival, we stratified the cohort by compliance with the Candida bundle. Supplementary Table 3 summarizes the adherence rates to each of the five Candida bundle components in the high-compliance and low-compliance groups. This revealed distinct patterns: in the high-compliance cohort, serum BDG status did not significantly affect survival (*p* = 0.66, log-rank test); however, in the low-compliance cohort, serum BDG positivity was significantly associated with decreased survival (*p* = 0.02, log-rank test) (Fig. 3). In the low-compliance group, the serum BDG-negative subgroup demonstrated no deaths. No significant differences were observed between the BDG-positive and BDG-negative subgroups with respect to age, sex, immunosuppressive agents, malignancy, and diabetes mellitus (Supplementary Table 4).

Regarding the secondary outcome, the incidence rate of Candida endophthalmitis was 27.9% (17/61) in the serum BDG-positive group and 15.8% (3/19) in the serum BDG-negative group, with no statistically significant difference (*p* = 0.37) (Table 3). Additionally, when comparing *C. albicans* with non-albicans species, the risk of developing endophthalmitis was significantly higher in patients with *C. albicans* infection (45.7% vs. 8.9%, *p* < 0.001). When stratified by Candida bundle adherence, the incidence of endophthalmitis was both 25.0% in the high-compliance group (18/72) and the low-compliance group (2/8). The median duration of antifungal therapy was significantly longer in patients with endophthalmitis compared to those without (45 days [IQR 31–89] vs. 21 days [IQR 16–39], respectively; *p* = 0.005).

**Table 3:** Ophthalmological findings by BDG status and *Candida* species

By BDG status	N = 96	BDG Positive (N = 70)	BDG Negative (N = 26)	*p* value
Ophthalmology consultation	80/96 (83.3)	61/70 (87.1)	19/26 (73.1)	0.13
Diagnosis of endophthalmitis	20/80 (25.0)	17/61 (27.9)	3/19 (15.8)	0.37

By Candida species	*Candida albicans* (N = 43)	non-albicans (N = 53)	*p* value
Ophthalmology consultation	35/43 (81.4)	45/53 (84.9)	0.78
Diagnosis of endophthalmitis	16/35 (45.7)	4/45 (8.9)	< 0.001

*BDG, (1, 3)-β-D-glucan

## Discussion

In this study, we evaluated the prognostic value of serum BDG levels in patients with candidemia, incorporating Candida bundle compliance scores. Among all patients with candidemia, serum BDG was negative in 27.1% of the patients. Mortality rates tended to be higher in patients with serum BDG-positive than those in the serum BDG-negative (21.4% vs. 7.7%; *p* = 0.09). Stratified analysis by Candida bundle compliance revealed that serum BDG positivity strongly indicated poor outcomes in the low-adherence group but not in the high-adherence group. The incidence of endophthalmitis was significantly higher in patients with *C. albicans* infection than in those with non-albicans species infection.

In BDG assay, both colorimetric and turbidimetric methods are established, with documented differences in their diagnostic sensitivity and specificity profiles [28]. In this study, all nine participating institutions commonly employed colorimetric methods. However, even within the subset of colorimetric assays, the optimal diagnostic threshold value demonstrates variability depending upon the reagent employed. In this study, we adopted the manufacturer-recommended cutoff values specific to each reagent system: 20 pg/mL for the Fungitec® G Test and 11 pg/mL for the Wako β-Glucan Test [27]. Although a serum BDG-negative status demonstrates a high negative predictive value for invasive fungal infections [29], evidence is conflicting [30, 31]. The frequency of serum BDG-negative candidemia ranges from 17.6 to 36% [24, 30], which is consistent with our findings. Several factors, including prior antifungal therapy [32], early BDG measurement post-onset [33], presence of hyperbilirubinemia or hypertriglyceridemia [34], and low fungal burden [24, 35], may contribute to false-negative serum BDG results. Detailed timings of antifungal therapy initiation were not available in our dataset. Therefore, we could not determine whether antifungal treatment preceded BDG measurement. Prior antifungal exposure may have led to decreased fungal burden and resulted in false-negative BDG results, potentially affecting the interpretation of our findings. Interestingly, higher rates of serum BDG negativity have been observed in patients infected with *C. glabrata* or *C. parapsilosis* [24, 36, 37]. BDG represents not merely a structural component of the fungal cell wall but also exists in substantial quantities within the biofilm extracellular matrix. Therefore, the lower serum BDG levels observed in *C. glabrata* may be attributed to its reduced capacity for biofilm production compared to *C. albicans.* Likewise, in *C. parapsilosis*, the relatively limited extracellular matrix composition within its biofilm structure may contribute to decreased BDG liberation into the bloodstream [38]. These interspecies variations in biofilm composition could potentially modulate serum BDG levels during candidemia episodes. However, our findings did not demonstrate this association. Instead, *C. guilliermondii* infection was significantly associated with serum BDG-negative results; this may potentially be attributable to its distinctive cell wall structure characterized by atypical mannan expression, which may attenuate host immune responses [39]. The relatively low incidence of *C. guilliermondii* infections in our cohort may limit the statistical power for definitive conclusions regarding its association with serum BDG negativity.

Previous studies have demonstrated an association between BDG negativity and better clinical outcomes [24]. Our findings indicate increased mortality in patients with serum BDG-positive, albeit not statistically significant. Due to the retrospective nature of the study and limitations in data availability, our analysis focused on the five components of the Candida bundle, which, however, potentially reflect the core aspects of candidemia management. When stratified by Candida bundle, BDG status demonstrated strong prognostic value in the low-compliance group, while exhibiting no statistically significant prognostic utility among patients in the high-compliance group. These findings suggest that the prognostic utility of BDG may be limited to patients with suboptimal Candida bundle compliance. This underscores that adherence to standardized management protocols is crucial, especially in patients with serum BDG-positive candidemia. We strongly insist that comprehensive adherence to the full Candida bundle—including other elements such as optimization of antifungal administration, clinical reassessment at early and appropriate intervals, and oral step-down therapy—remains ideal in routine clinical practice and should be encouraged whenever feasible.

Candida endophthalmitis complicates approximately 15–25% of patients with candidemia, with *C. albicans* infection and CVC insertion constituting established risk factors [7, 40, 41]. Our analysis corroborates a significant association between *C. albicans* infection and increased incidence of endophthalmitis. While studies have suggested elevated serum BDG levels (> 108 pg/mL, Wako assay) predict ocular involvement [7], we observed no significant correlation between serum BDG status and the development of endophthalmitis. These findings highlight the requirement of comprehensive ophthalmological evaluation in all patients with candidemia, regardless of serum BDG results, with particular vigilance warranted in patients with *C. albicans* infection.

This is the first study to evaluate the prognostic utility of BDG in candidemia stratified by Candida bundle adherence, with data collected across nine hospitals in Japan. However, this study has certain limitations to be addressed. First, the retrospective study design inherently constrained data collection, necessitating us to focus on five key bundle components rather than the complete nine-component protocol. We excluded four potential components as follows: (1) early clinical reassessment, (2) accurate initial dosing, (3) appropriate alternative selection, and (4) oral step-down therapy. Delayed clinical reassessment, inadequate dosing, and inappropriate choice of antifungal agents for patients with drug-resistant strains could adversely affect patients’ prognosis; therefore, adherence to these practices should be thoroughly investigated. However, validating these points retrospectively is highly complex and rarely feasible, requiring far more dedicated efforts and a comprehensive dataset. Oral step-down antifungal therapy was excluded from the evaluation in the present study, as its impact on patient outcomes has not been shown to be consistent. The inclusion of patients who received oral step-down antifungal therapy may also have introduced variability in illness severity and treatment intensity. Omission of these elements could have certainly influenced the evaluation of bundle adherence and outcomes of the present study. Second, the limited sample size, particularly in subgroup analyses, surely reduced the statistical power to detect significant associations. For example, while serum BDG positivity was associated with numerically higher 30-day mortality (21.4% vs. 7.7%), the difference did not reach statistical significance in the overall cohort, possibly representing a Type II error as a result of smaller sample sizes. Only 16 patients were included in the low bundle adherence group, limiting the interpretability of interaction effects between BDG status and bundle adherence. This reduction in sample size and the strict inclusion criteria also have implications for generalizability. In the absence of formal sensitivity analysis, the observed outcomes indicate the need for future prospective studies to validate these findings in larger, more diverse populations with predefined power analyses. Third, although two commercial BDG assays were used—Fungitec® G and Wako—both commonly employ the colorimetric method. Using their respective cutoffs (20 pg/mL for Fungitec® G; 11 pg/mL for Wako), both assays demonstrate comparable sensitivity. As most patients were tested with the Fungitec® G assay, inter-assay variability likely had minimal impact. Importantly, no turbidimetric assays—which generally have lower sensitivity—were used, possibly contributing to a higher proportion of BDG-positive cases compared to studies using such methods. Fourth, the absence of standardized adjustment for patient backgrounds and characteristics constrained our capacity to evaluate the influence of disease severity on clinical outcomes. We propose that future prospective studies should incorporate standardized severity scoring systems and adjust for relevant clinical covariates to more accurately assess the independent prognostic significance of serum BDG levels and adherence to the Candida bundle. Nevertheless, these findings provide novel insights into the relationship between serum BDG status, Candida bundle compliance, and clinical outcomes in candidemia.

## Conclusion

In summary, serum BDG status may have prognostic value, especially in patients with suboptimal compliance to the Candida bundle. The findings suggest that strict compliance with the treatment protocol is particularly crucial in patients with serum BDG-positive candidemia. Also, ophthalmological evaluation is necessary for all patients with candidemia regardless of serum BDG levels.

## Supplementary Information

Below is the link to the electronic supplementary material.

Supplementary file1 (DOCX 23 kb)

## Data Availability

Detailed data are available upon request from the corresponding author.
