# Supplementary material for: Prognostic Value of Serum (1→3)-β-D-Glucan Levels in Patients with Candidemia Stratified by Compliance with Candida Bundle: A Multicenter Retrospective Cohort Study (2016–2023)
Source: Mycopathologia. 2025 Sep 22;190(6):90. doi: 10.1007/s11046-025-00999-7 (PMC12454490; doi:10.1007/s11046-025-00999-7)
Supplement: Supplementary file 1 — Supplementary file1 (DOCX 23 kb) [file 11046_2025_999_MOESM1_ESM.docx]

**Supplementary Table 1. Blood culture system of each facility**

|  | Blood culture bottle | Blood culture device | Days of culture |
| --- | --- | --- | --- |
| ―Okayama University Hospital  ―Tsuyama Chuo Hospital  ―Okayama Medical Center  ―Okayama Rousai Hospital  ―Okayama Red Cross Hospital  ―Takahashi Central Hospital  ―Kurashiki Medical Center  ―Tottori Municipal Hospital  ―Marugame Medical Center | BD BACTEC  BACT/ALERT  BD BACTEC  BD BACTEC  BACT/ALERT  BD BACTEC  Signal Blood Culture system  BD BACTEC  BD BACTEC | BD BACTEC FX  BACT/ALERT VIRTUO  BD BACTEC FX  BD BACTEC FX  BACT/ALERT VIRTUO  BD BACTEC FX  Signal Blood Culture system  BD BACTEC FX  BD BACTEC FX | 7  5  7  5  7  7  7  7  7 |

**Supplementary Table 2. Serum BDG assay methodology of each hospital**

|  | BDG measurement reagents | BDG assay equipment | BDG assay method |
| --- | --- | --- | --- |
| ―Okayama University Hospital  ―Tsuyama Chuo Hospital  ―Okayama Medical Center  ―Okayama Rousai Hospital  ―Okayama Red Cross Hospital  ―Takahashi Central Hospital  ―Kurashiki Medical Center  ―Tottori City Hospital  ―Marugame Medical Center | Fungitec® G Test  Fungitec® G Test  Fungitec® G Test  Fungitec® G Test  Wako β-Glucan Test  Fungitec® G Test  Fungitec® G Test  Fungitec® G Test  Fungitec® G Test | ES Analyzer  Wellreader SK603  Wellreader SK603  ES Analyzer  LIMUSAVE MT-7500  ES Analyzer  Wellreader SK603  Wellreader SK603  Wellreader SK603 | colorimetric synthetic substrate method colorimetric synthetic substrate method colorimetric synthetic substrate method colorimetric synthetic substrate method colorimetric synthetic substrate method colorimetric synthetic substrate method colorimetric synthetic substrate method colorimetric synthetic substrate method colorimetric synthetic substrate method |

＊Abbreviations: BDG, (1, 3)-β-D-glucan

**Supplementary Table 3. Adherence rates to each Candida bundle component in the high-compliance and low-compliance groups**

|  | **All patients**  **(N=96)** | **High-compliance**  **(N=80)** | **Low-compliance**  **(N=16)** |
| --- | --- | --- | --- |
| CVC removal, N (%) | 70 (72.9) | 63 (78.8) | 7 (43.8) |
| Appropriate antifungal agent, N (%) | 87 (90.6) | 76 (95.0) | 11 (68.8) |
| Ophthalmological evaluations, N (%) | 80 (83.3) | 75 (93.8) | 5 (31.3) |
| Blood culture monitoring until clearance, N (%) | 84 (87.5) | 75 (93.8) | 9 (56.3) |
| Antifungal therapies for a minimum of two weeks following candidemia clearance, N (%) | 83 (86.5) | 76 (95) | 7 (43.8) |

**Supplementary Table 4. Clinical characteristics of the low-compliance Candida bundle group by BDG status**

|  | **BDG Positive**  **(N=11)** | **BDG Negative**  **(N=5)** | *p* value |
| --- | --- | --- | --- |
| Age, median [interquartile range] | 74 [54–76] | 68 [60–81] | 0.57 |
| Sex, male, N (%) | 6 (54.5) | 5 (100) | 0.12 |
| Immunosuppressive agents, N (%) | 7 (63.6) | 2 (40) | 0.60 |
| Malignancy, N (%) | 8 (72.7) | 3 (60) | 1.00 |
| Diabetes mellitus, N (%) | 3 (27.3) | 4 (80) | 0.11 |
